# Delivering the evidence to improve the health of women and newborns: State of the World’s Midwifery, report 2014

**DOI:** 10.1186/1742-4755-11-89

**Published:** 2014-12-17

**Authors:** Frances Day-Stirk, Frances McConville, James Campbell, Laura Laski, Maria Guerra-Arias, Petra ten Hoope-Bender, Michaela Michel-Schuldt, Luc de Bernis

**Affiliations:** Int. Conf. of Midwives (ICM), Laan van Meerdervoort 70, 2517 AN The Hague, The Netherlands; World Health Organization (WHO/MCA), Avenue Appia 20, 1211 Geneva 27, Switzerland; World Health Organization (WHO/HWF), Avenue Appia 20, 1211 Geneva 27, Switzerland; United Nations Population Fund (UNFPA), 605 Third Avenue, New York, New York 10158 USA; ICS Instituto de Cooperación Social Integrare, calle Balmes 30, 3º-1, 08007 Barcelona, Spain; United Nations Population Fund (UNFPA),, International Environment House, 11, Chemin des Anémones, CH-1219 Châtelaine, Geneva, Switzerland

## Abstract

The State of the World’s Midwifery Report 2014: A universal pathway, a women’s right to health (SoWMy2014) was published in June 2014 and joins the ranks of a number of publications which contribute to the growing body of evidence about a global midwifery workforce that can improve maternal and child health.

This editorial provides an overview of these publications that have been supported by global movements in the area of sexual, reproductive, maternal, and newborn and child health over the last four years. Background information is given on the methodology and data collection of SoWMy2014, the main findings cover the area of the availability, accessibility, acceptability and quality of midwifery services and a 2 page country brief shows the SRMNH data and workforce projections for each of the 73 “Countdown countries” that participated.

SoWMy 2014 report shows that midwives can provide 87% of the needed essential care for women and newborns, when educated and trained to international standards. Midwives however, are most effective when they work within a functional health system and enabling environment.

Also, a supportive team of auxiliaries, physicians and specialists is essential in order to ensure coverage of SRMNH services to women and newborns across the whole continuum of care, from pre-pregnancy through to pregnancy, childbirth and the post-natal period and from household to hospital.

Based on these findings, the report puts forward a vision of Midwifery2030, a pathway for women’s health and for midwifery policy and planning through the end of 2030. It promotes women-centered and midwife-led care to achieve the goal of universal health coverage for all women.

**Electronic supplementary material:**

The online version of this article (doi:10.1186/1742-4755-11-89) contains supplementary material, which is available to authorized users.

## Commentary

The past four years have seen the global evidence base on the midwifery workforce and midwifery services grow and develop to a previously unprecedented extent. In 2010 the first Global Midwifery Symposium, under the leadership of the United Nations Population Fund (UNFPA) and the International Confederation of Midwives (ICM), was held in Washington DC, and issued a Global Call to Action to strengthen midwifery services [1]. In response to this call to action, *The State of the World’s Midwifery: Delivering Health, Saving Lives*, was published in 2011 [2]. It provided the first comprehensive overview of the availability and quality of midwifery services around the world, profiling 58 countries with high rates of maternal and newborn mortality.

This momentum continued with the Second Global Midwifery Symposium (Kuala Lumpur, 2013) where the groundwork was laid for the *State of the World’s Midwifery Report 2014: A universal pathway, a women’s right to health*[3], published the following year*.* The 75 countries of the *Countdown to 2015* initiative [4] were invited to participate in the *SoWMy 2014* report, of which 73 responded to the request.

At the same time, 2014 saw the publication of the Lancet Series on Midwifery, which provides a rigorous scientific grounding and a framework for Quality Maternal and Newborn Care that identifies what should be done to improve maternal and newborn health, as well as how and by whom.

In addition, Midwifery workforce assessments, supported by the H4+ High Burden Countries Initiative (HBCI), have been conducted or are ongoing in six of the countries with high numbers of maternal and newborn deaths: Afghanistan, Bangladesh, Tanzania, DRC, Mozambique and Ethiopia. This methodology has also been used in smaller countries, including Benin, Guinea and Togo. These in-depth assessments aim to identify gaps in service availability, quality and accessibility, and support national authorities to develop costed implementation strategies, tailored to the needs and capacities of each country. An operational guidance, which will allow Ministries of Health and other national stakeholders to replicate these assessments in their country context, is currently being developed by UNFPA and will be published in 2014.

This trajectory of research has been aligned with key international movements supporting advances in maternal and newborn health, such as the UN Secretary General’s *Every Woman, Every Child* (EWEC) initiative, Saving Mothers, Giving Life [5]; the Campaign for Accelerated Reduction of Maternal Mortality in Africa (CARMMA) [6]; UNICEF’s A Promise Renewed to end preventable child deaths [7]; and the Family Planning 2020 movement (FP2020) [8]. Recognising the value and impact of midwifery services, a number of countries have decided to invest in midwives and other health professionals for SRMNH, and significant progress is becoming visible in many.

A number of past and current programmes, including the ICM-UNFPA Investing in Midwifery programme [9], funded by several donors; the World Bank Sahel Women’s Empowerment and Demographics Project [10]; the WHO/AFRO new Training curricula [11]; and the new ASEAN Guideline for Training and Accreditation of Skilled Birth Attendants [12], are assisting governments in strengthening plans, education and training programmes and regulations. In addition to the Global Standards for Midwifery Education and Regulation and the Essential Competencies for Basic Midwifery Practice, [13] another important initiative being created by the ICM is the Guiding Framework for the development of SRMNH services by midwives, to help governments and development partners develop or strengthen midwifery services from within their national context and situation.

With updated evidence and analysis on the availability, accessibility, acceptability and quality of midwifery services SoWMy 2014 shows how the body of evidence for midwifery care is advancing in leaps and bounds [14].

The essential finding of the *SoWMy 2014* report (Additional file 1) is that midwives can provide 87% of the needed essential care for women and newborns, when educated and trained to international standards. Midwives however, are most effective when they work within a functional health system and enabling environment. And a supportive team of auxiliaries, physicians and specialists is essential in order to ensure coverage of SRMNH services to women and newborns across the whole continuum of care, from pre-pregnancy through to pregnancy, childbirth and the post-natal period and from household to hospital.

With this in mind, the report puts forward a vision of *Midwifery2030*, a pathway for midwifery policy and planning to the end of the 2030 period which promotes women-centred and midwife-led models of care to achieve the goal of universal health coverage for all women. In details, Midwifery2030 suggests 10 pillars:

1. All women of reproductive age, including adolescents, have universal access to midwifery care when needed (the first and second components of UHC).
2. Governments provide and are held accountable for a supportive policy environment.
3. Governments and health systems provide and are held accountable for a fully enabled environment.
4. Data collection and analysis are fully embedded in service delivery and development.
5. Midwifery care is prioritized in national health budgets; all women obtain universal financial protection (the third component of UHC).
6. Midwifery care is delivered in collaborative practice with health-care professionals, associates and lay health workers.
7. First-level midwifery care is close to the woman and her family with seamless transfer to next-level care.
8. The midwifery workforce, in communities, facilities and hospitals, is supported through quality education, regulation and effective human and other resource management.
9. All health-care professionals provide, and are enabled to deliver respectful, quality care.
10. Professional associations provide leadership to their members to facilitate quality care through advocacy, policy engagement and collaboration.

One of the goals highlights the need for more accurate, up-to-date and disaggregated data on the midwifery workforce, across the effective coverage domains of availability, accessibility, acceptability and quality [15]. Although great progress has been made since the first report in 2011, with many countries reporting advances in data information systems for human resources for health, there is still a long way to go. The *SoWMy 2014* report shows that just 10 minimum pieces of information can help policy makers to effectively plan for the future, taking into account the likely evolution of the population. These are: headcount, percentage time spent on SRMNH, roles, age distribution, retirement age, length of education, enrolments into, attrition and graduation from education, and voluntary attrition from the workforce. However, the availability of this information in the *SoWMy 2014* survey shows considerable gaps. For example, information on headcount and percentage time spent on SRMNH was available for about 90% of the health worker cadres reported by countries, but information on the age distribution and attrition from the workforce was available for only about 40% of reported cadres. Likewise, reliable information on the number of student enrolments or graduates in 2012 was available for less than 60% of the reported cadres. Ensuring that this vital information is collected in future will require strengthening the capacity of national departments for human resources for health, thus allowing ministries to make informed decisions on the challenges faced in country.

These data, along with other key indicators on the demographic and epidemiological characteristics of the population, inform the 73 country briefs of the *SoWMy2014* report. These include information on current workforce availability and met need for SRMNH services, as well as its projected trajectory to 2030 based on demographic changes and the evolution of the workforce accounting for both inflows and outflows. For the African region, which has the highest rates of maternal and newborn mortality in the world, analysis of the *SoWMy2014* briefs allows for the identification of several common patterns within countries. In some, high population growth rates and lack of resources to invest in human resources for health result in dismal met need of the population, which unfortunately is not projected to improve in the near future (e.g. Burkina Faso, Central African Republic, Chad, Congo, Mali). Others may see their current high met need situation deteriorate in the next decade, as high fertility rates increase the need for services, while at the same time retirement of an ageing workforce leads to losses which are not addressed by investment into the production of new health workers (e.g. Democratic Republic of the Congo, Zambia, Rwanda). In another group of countries, estimates of high levels of met need for SRMNH services may not correspond with the reality on the ground, if for example, inadequate deployment mechanisms, geographical or financial barriers prevent equitable access to the health care professionals (e.g. Liberia, Nigeria, Zimbabwe). Finally, the briefs reveal a group of countries which are currently on a good track towards improving the met need of SRMNH services in the population, providing that they keep training new health workers, as projected (e.g. Angola, Cote d’Ivoire, Ghana, Kenya, Malawi).

Additionally, accurate data can enable countries to model alternative policy scenarios and observe the impact on meeting the population’s need for SRMNH services. Conducting these projections for 73 countries, the *SoWMy2014* report shows that average estimated met need in 2030 could rise from 60%, under the current trajectory, to 87%, with concerted policy actions. These include efforts to reduce pregnancies, increase the number of midwife, nurse and physician graduates, improve the efficiency of SRMNH workers, and reduce leakages from the workforce due to attrition.

By taking ownership of their SoWMy data and national briefs, national stakeholders can set the foundations for an effective policy dialogue. Country ownership, capacity building and dialogue have been essential components and aims of the *SoWMy 2014* project since its conception stage, to ensure that the findings and recommendations of the report are used to generate real transformative impact. As well as the 37 stakeholder workshops held during the data collection process, this process is ongoing with the national launches of the report, led by ministries of health with the support of UNFPA, WHO and ICM country teams, the latter acting through national midwives associations, and engaging the support of other key stakeholders involved at the individual country level (including Jhpiego, White Ribbon Alliance, Family Care International, Evidence for Action, and others and with Johnson & Johnson support). As of September 2014, 11 launches have taken place, generating broad media coverage and policy commitments from national ministries [16–20] and up to 25 more are projected for the coming months. The launches can also serve to strengthen the capacity of local actors, such as national midwives associations, providing them with a platform in which to engage with high level stakeholders.

Although the country briefs show many challenges, and the alternative policy trajectory is very ambitious, with political prioritisation, international support, and using proven best practices which have demonstrated success in other contexts, the alternative can be within the reach of all countries committed to expanding universal coverage of essential SRMNH services to the population, and meeting international targets to end, once and for all, the tragedy of preventable maternal and neonatal deaths.

## Electronic supplementary material

Additional file 1: Midiwifery 2030, a pathway to health. (JPEG 946 KB)
