# Supplementary figures and images for: Delivering the evidence to improve the health of women and newborns: State of the World’s Midwifery, report 2014
Source: Reprod Health. 2014 Dec 17;11:89. doi: 10.1186/1742-4755-11-89 (PMC4326403; doi:10.1186/1742-4755-11-89)

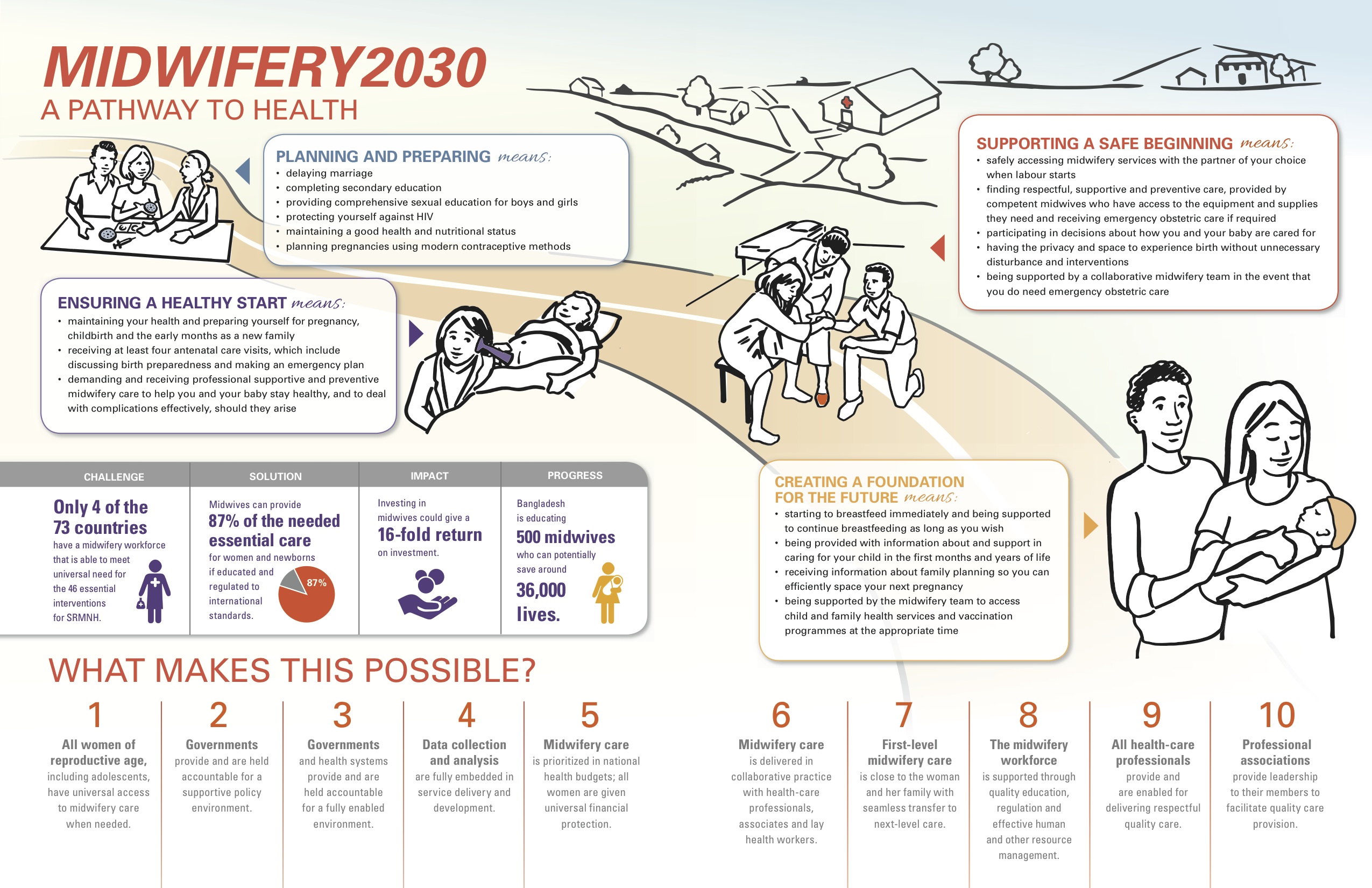

Supplement: Supplementary file 1 — Additional file 1: Midiwifery 2030, a pathway to health. (JPEG 946 KB) [file 12978_2014_349_MOESM1_ESM.jpeg]
